# Metabolic engineering of *Komagataella phaffii* for enhanced production of heterologous proteins and small molecules: key strategies and future directions

**DOI:** 10.1128/aem.00110-26

**Published:** 2026-07-16

**Authors:** Jiaoyu Xin, Lu Zhao, Youxin Meng, Ziye Tian, Kaikai Lv, Chenyu Xue, Na Dong

**Affiliations:** 1 Laboratory of Molecular Nutrition and Immunity, College of Animal Science and Technology, Northeast Agricultural University 12430, Harbin, People's Republic of China; The Pennsylvania State University, University Park, Pennsylvania, USA

**Keywords:** *Komagataella phaffii*, *Pichia pastoris*, heterologous proteins, small molecules, biosynthetic pathways, metabolic engineering

## Abstract

*Komagataella phaffii (syn. Pichia pastoris*) is an important host for the production of recombinant proteins and small molecules, often exhibiting superior performance compared to other heterologous hosts. With advancements in synthetic biology and computational biology, metabolic engineering has become a key strategy for achieving high-level heterologous proteins and small molecules in *K. phaffii* for industrial and commercial applications. In this review, we provide an overview of advances in *K. phaffii* metabolic engineering. We first discuss the development of the *K. phaffii* expression system. Subsequently, we analyze core optimization strategies, including metabolic flux control, metabolic dynamic control, application of system modeling, chassis cell optimization, and recombinant protein and small-molecule production, before finally summarizing the current challenges and future development directions.

## INTRODUCTION

*Komagataella phaffii* (synonymous with *Pichia pastoris*), initially identified in the exudate of French chestnut trees, was originally designated *Zygosaccharomyces pastoris* by the mycologist and cytologist Alexandre Guilliermond (1). In the 1950s, Phaff et al. reclassified it into the genus *Pichia*, renaming it *Pichia pastoris*, a name that subsequently saw widespread use in both research and industrial applications (2). At the end of the 20th century, based on ribosomal RNA sequence analysis, Yamada et al. proposed the new genus *Komagataella*, incorporating the related methanol-assimilating yeasts into this group (3, 4). Therefore, the established name, *Pichia pastoris*, will be used synonymously to refer to the engineered strains of the *Komagataella* genus solely in the context of biotechnological applications. In its initial applications, *K. phaffii* was employed by Phillips Petroleum Company for commercial-scale production, leveraging methanol in high-cell-density fermentation processes to yield single-cell protein (SCP) for animal feed supplementation. Subsequently, it was decided to release it to laboratories for academic research, leading to the rapid development of the *K. phaffii* expression system (5). *K. phaffii*’s inherent tolerance to elevated temperatures, acidic or alkaline conditions, and high methanol concentrations has rendered it a focal point of considerable interest in contemporary biotechnological applications.

Metabolic engineering strategies in *K. phaffii* are designed to optimize its metabolic network via genetic engineering and metabolic flux regulation, thereby enhancing the large-scale production of heterologous proteins and a wide array of small molecules. In recent years, the application of high-throughput omics technologies, including genomics, transcriptomics, and metabolomics, has facilitated the identification of key genes within the *K. phaffii* metabolic network and enabled the use of synthetic biology approaches to engineer its biosynthetic pathways (6). Nevertheless, the exponential increase in data volume can introduce analytical complexities, resulting in a “data deluge” that challenges traditional methods. Consequently, both metabolic modeling and machine learning (ML) hold significant potential for supporting metabolic engineering efforts (7, 8). Currently, the main synthetic biology tools for engineering *K. phaffii* include heterologous gene expression vectors, promoters, terminators, and CRISPR/Cas systems (9). Specifically, CRISPR-Cas systems enable the creation of thousands of targeted mutations throughout metabolic pathways (10). Advancements in synthetic biology and computational biology have fostered promising avenues for employing metabolic engineering in *K. phaffii* to synthesize commercially valuable compounds.

Building on previous reviews (11–13), the present review synthesizes recent progress in the metabolic engineering of *K. phaffii*. This review begins by tracing the evolutionary trajectory of the expression systems associated with this yeast strain, with a particular emphasis on genetic engineering technologies. It then elaborates on the key strategies for optimizing the metabolic engineering of *K. phaffii*. These encompass metabolic flux regulation, dynamic metabolic control, the application of systems modeling, chassis cell optimization, and the production of recombinant proteins and small molecules. Subsequently, the practical applications of this strain in agriculture, industry, food, and pharmaceutical sectors are detailed. The paper concludes by discussing current research challenges and future directions. This review underscores that the core elements for fully realizing the potential of *K. phaffii* metabolic engineering involve the optimization of metabolic fluxes through rational, targeted modification techniques, enhanced collaboration with computational disciplines, and multi-scale engineering design and optimization spanning the molecular, cellular, and systems levels.

## DEVELOPMENT OF THE *K. PHAFFII* EXPRESSION SYSTEM

Proteins, which are biomolecules comprised of amino acids, execute a diverse array of functions within living organisms. However, naturally sourced proteins often suffer from limited availability, insufficient purity, and high production costs. To circumvent these limitations, *Escherichia coli* was initially utilized as a host organism for the production of recombinant insulin during the 1980s (14). However, as a prokaryotic organism, *E. coli* encounters challenges in recombinant protein expression, such as the formation of inclusion bodies composed of misfolded proteins (15). These inclusion bodies can hinder the downstream purification of the recombinant protein. Therefore, the selection of an appropriate host cell is a critical factor for the successful and efficient expression of recombinant proteins.

*K. phaffii*, a eukaryotic and methylotrophic yeast, possesses the ability to utilize methanol as its sole carbon and energy source (16). In addition to its capacity for high-cell-density growth in simple media, *K. phaffii* can also thrive in dynamic culture systems, where variables such as dissolved oxygen levels, pH, glucose concentration, and temperature must be carefully controlled (17). Furthermore, the *K. phaffii* expression system offers advantages, including post-translational modifications, genetic stability, disulfide bond formation, and glycosylation (18–21). Its mature extracellular protein secretion system, combined with the guidance of the α-mating factor (MF) signal sequence, enables *K. phaffii* to achieve high-yield secretion of heterologous proteins. This efficient targeting dramatically minimizes host-derived background contaminants, consequently enhancing the solubility of the secreted product and streamlining its purification (22). Owing to the distinctive advantages of its expression system, *K. phaffii* is increasingly becoming one of the preferred microbial hosts for the production of recombinant proteins and small molecules (18).

For decades, researchers have continuously developed new genetic tools to overcome limitations in both research and commercial applications, aiming to construct highly adaptable and productive *K. phaffii* strains. In 1985, *K. phaffii* was initially developed as a heterologous protein expression system after the pioneering application of the methanol-inducible *AOX1* promoter (*P_AOX1_*), which is tightly regulated by methanol (23). However, the *P_AOX1_* has drawbacks, such as methanol toxicity and leaky expression of target genes, thereby limiting its application scope. To address this, the native *GAP* promoter (*P_GAP_*) was used, which, compared to *P_AOX1_*, can achieve efficient expression in the absence of methanol. Following transcriptional regulation, enhancing the efficiency of translation and secretion becomes the next critical juncture. To this end, multiple strategies have been widely implemented. These include codon optimization to increase the rate and fidelity of translational elongation (24, 25); replacement of inefficient native signal peptides with sequences that are efficiently recognized and cleaved by *K. phaffii* to drive high-yield protein secretion (26, 27); and high-throughput screening for strains exhibiting the highest secretory capacity and the lowest background of host proteases, thereby avoiding protein degradation and alleviating cellular burden (28). Collectively, these strategies constitute an effective means to improve the expression level of heterologous proteins. Studies have demonstrated that significantly enhanced α-galactosidase expression in *K. phaffii*, achieving an extracellular activity of 1,299 U/mL through strategies including codon optimization, replacement of the native signal peptide sequence with the *Saccharomyces cerevisiae* α-factor signal peptide, and selection of an optimal host strain (29). Rong et al. (30), through a combination of strategies including identifying the optimal expression cassette (*P_AOX1_* with SP_0030_ signal peptide), optimizing gene copy number, overexpressing protein disulfide isomerase (PDI), and systematically refining fermentation parameters, achieved a final brazzein yield of 639 mg/L. Condition optimization is also an effective strategy to enhance heterologous protein and small-molecule expression levels. For example, this includes controlling methanol concentrations, feeding frequency, and co-feeding supplementary carbon sources (31).

Moreover, it is particularly noteworthy that gene editing can effectively and precisely modify and engineer the *K. phaffii* genome, offering a customized host expression system for recombinant proteins and small molecules. In *K. phaffii*, most targeted gene knockouts or knock-ins have been achieved using non-homologous end joining (NHEJ). NHEJ pathway repairs DNA double-strand breaks (DSBs) by directly ligating the broken ends without requiring a homologous template. This makes it a straightforward and efficient repair mechanism. However, it easily introduces small insertion or deletion mutations, which may cause abnormal function of the target gene or increased genomic instability (32). In contrast to NHEJ, the homologous recombination (HR) pathway utilizes a homologous DNA template for high-fidelity repair. This mechanism not only enables precise gene knockout or knock-in screening but also contributes to enhanced genomic stability (33, 34). While HR efficiency in *K. phaffii* is lower than that in *S. cerevisiae*, this bottleneck has been overcome through technical strategies such as double-gene knockout and transient suppression of the homologous gene encoding Ku70, a key DNA end-binding protein in the NHEJ pathway (35).

In recent years, CRISPR/Cas9, a well-established gene editing technology, has been explored for gene modification in *K. phaffii*. By utilizing a single-guide RNA (sgRNA) plasmid containing an autonomously replicating sequence (ARS) to direct Cas9, a DSB is introduced at the target genomic locus. The subsequent editing efficiency is then primarily dependent on the HR repair pathway (36). CRISPR/Cas9 genome editing, driven by the SER promoter for sgRNA expression, has been reported to demonstrate a 10-fold enhancement in editing efficiency, attributed to the plasmid stability conferred by panARS (37). In addition, serial passaging can be employed to eliminate residual sgRNA, thereby enabling complete (100%) genome editing of single-gene targets (38). To further enhance HR efficiency, Gao et al. achieved single-step multi-locus integration by co-overexpressing the exogenous HR key genes *RAD52*, *RAD59*, and *MRE11* from *S. cerevisiae* with 40 bp short homology arms. This strategy increased the triple-locus integration efficiency from 45% to 81%, representing a 4.3-fold improvement in HR efficiency (39). Separately, Bai et al. established a tetracycline repressor (TetR)/tetO₂ induction system to regulate the expression of HR-related genes *RAD52* and *MUS81-MMS4*. This system boosted the positive rate for single-gene knockout and the assembly efficiency for multiple loci to 81%, without compromising subsequent cell growth or product synthesis (34). However, existing gene-editing approaches mostly rely on the DSB repair pathway, which is prone to insertions and deletions (indels) and makes precise point mutations difficult to achieve. To overcome the limitations of traditional DSB editing, Wu et al. (40) developed base-editing (BE) tools based on the CRISPR-Cas9 system and screened for the optimal BE construct (PAOX2*-KpA3A-nCas9-KpUGI-DAS1TT), achieving a C-to-T editing efficiency of up to 96.0% and an editing window of 7 nucleotides. Furthermore, by replacing nCas9 with nSpG and nSpRy variants, which have broader protospacer adjacent motif (PAM) compatibility, efficient targeting was achieved at NGN and NRN-PAM sites, respectively, significantly broadening the scope of genome editability. Additionally, by integrating T7 RNA polymerase (T7 RNAP) with the CRISPRa system, inserting a transfer RNA (tRNA) sequence between the T7 promoter and the sgRNA increased the activation efficiency 4.0-fold and allowed for simultaneous multi-gene activation. Based on this, an AND logic gate was constructed, achieving 3.3-fold targeted activation under dual induction by methanol and doxycycline, alongside an OR logic gate, in which dual induction yielded a synergistic efficiency of 3.42-fold compared to single-induction activation. Furthermore, developing an Unfolded Protein Response (UPR) self-responsive system enabled the dynamic regulation of the endogenous transcription factor HAC1, ultimately leading to a patatin yield of 353 mg/L, a 116% increase over that of the original strain (41).

With increasingly refined exploration at the molecular level, multi-omics technologies have become pivotal tools for life science researchers. Since 2009, whole-genome sequencing has been completed for several representative strains of *K. phaffii*, including GS115, DSMZ 70382, and CBS7435 (42–44). Building upon this genomic foundation, a series of transcriptomic, proteomic, and metabolomic studies have subsequently been conducted, laying the groundwork for a deeper understanding of the advantages and limitations of *K. phaffii* as a chassis organism for heterologous protein expression and metabolite synthesis (45–47). On this basis, and in conjunction with advances in novel genetic tools and synthetic biology, technical approaches such as glycoengineering, metabolic flux analysis, high-throughput screening, metabolic engineering, and mathematical modeling have collectively propelled the *K. phaffii* expression system toward breakthrough applications across diverse fields, including agriculture, industry, food, and pharmaceuticals (26, 28, 48–50).

## KEY STRATEGIES IN *K. PHAFFII* METABOLIC ENGINEERING

*K. phaffii*, as an excellent cell factory, has seen the evolution of its metabolic engineering strategies from static and localized optimizations toward dynamic, systems-level frameworks. Optimization has gradually extended from static manipulation of core metabolic routes (e.g., carbon, nitrogen, and energy fluxes) to dynamic metabolic regulation capable of autonomous homeostasis maintenance and balancing of growth and product formation, thereby overcoming the inherent constraints of static control mechanisms. With the development of synthetic biology and systems biology technologies, systems metabolic engineering involves the holistic design and reprogramming of the cell, integrating organically with chassis strain optimization to build highly efficient cell factories with multi-dimensional synergy, becoming the core direction of current metabolic engineering. Meanwhile, specialized optimization techniques have been developed for critical steps in recombinant protein production, such as glycosylation modification and endoplasmic reticulum (ER) processing. Moreover, this technological advancement is simultaneously broadening its scope to encompass the synthesis of small molecules derived from renewable feedstocks.

### Metabolic optimization

#### Optimization of metabolic flux

During the high-density fermentation of *K. phaffii*, nutrient and energy provisioning is a critical step for enhancing yeast growth rate and metabolic activity. Methanol, glucose, glycerol, and ammonium sulfate are commonly used as carbon and nitrogen sources in the fermentation process. Carbon source metabolism in *K. phaffii* encompasses glycerol metabolism, methanol metabolism, sugar metabolism, and lipid metabolism, while nitrogen source metabolism is achieved through the interconversion of ammonia, glutamate, and glutamine, facilitating metabolic regulation. Furthermore, energy metabolism functions as an indispensable synergistic modulator within this process. Consequently, understanding and optimizing these metabolic processes are essential for efficient fermentation.

##### Optimization of carbon metabolism

**Fig 1:**
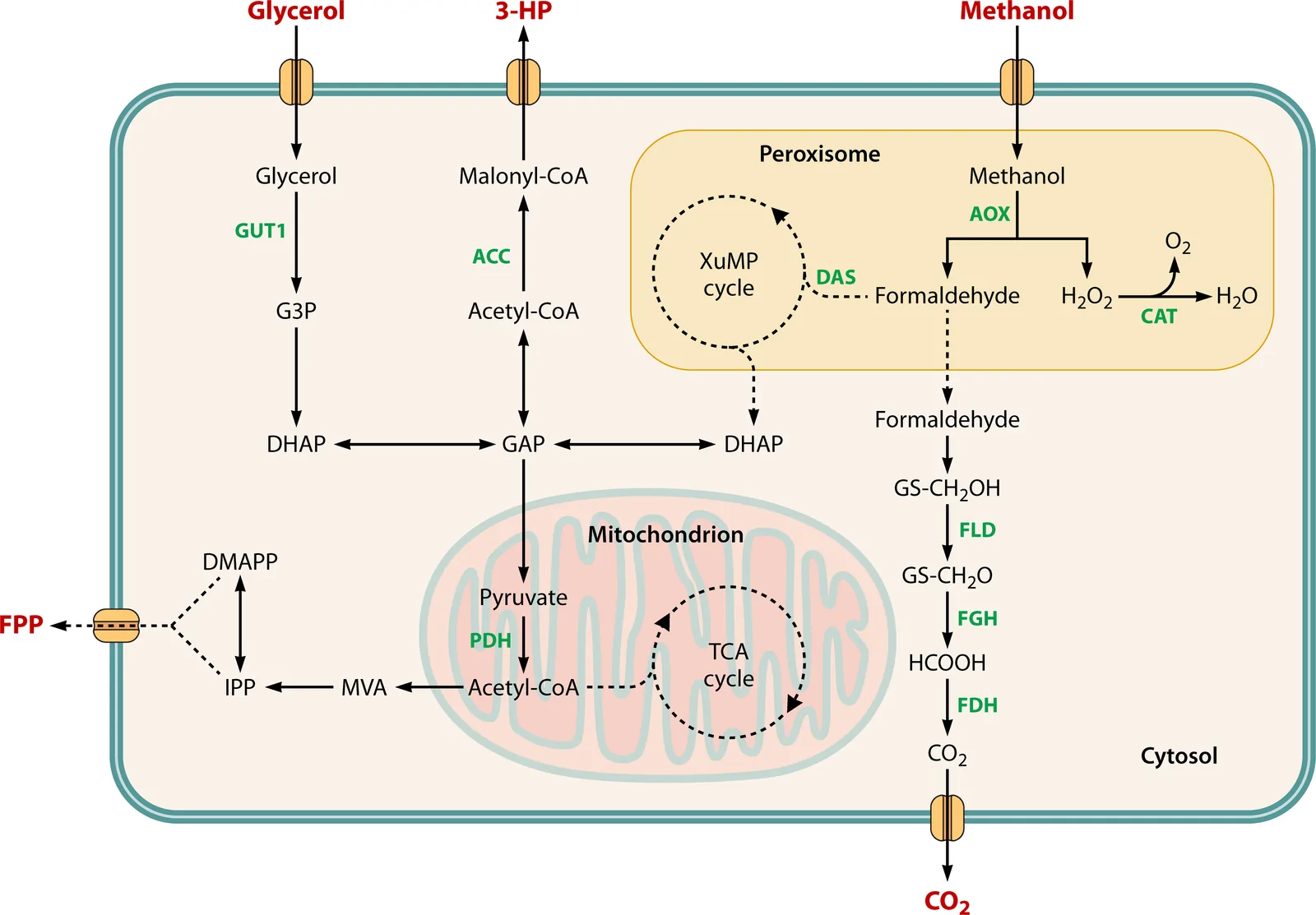
Carbon metabolism in *K. phaffii*. GUT1, glycerol kinase; G3P, glycerol-3-phosphate; DHAP, dihydroxyacetone phosphate; GAP, glyceraldehyde-3-phosphate; AOX, alcohol oxidase; CAT, catalase; DAS, dihydroxyacetone synthase; FLD, formaldehyde dehydrogenase; FGH, S-formylglutathione hydrolase; FDH, formate dehydrogenase; XuMP, xylulose monophosphate pathway; MVA, mevalonate; IPP, isopentenyl pyrophosphate; DMAPP, dimethylallyl pyrophosphate.

The fermentation process of *K. phaffii* is divided into a glycerol growth phase and a methanol induction phase. In the glycerol growth phase, *K. phaffii* relies on glycerol as its sole carbon source, which is then metabolized through the glycerol metabolic pathway. Glycerol, under the catalysis of glycerol kinase (GUT1), produces glycerol-3-phosphate (G3P), ultimately leading to the formation of dihydroxyacetone phosphate (DHAP) and glyceraldehyde-3-phosphate (GAP). The latter can enter the Embden-Meyerhof-Parnas (EMP) pathway to form pyruvate, which then enters the tricarboxylic acid (TCA) cycle (Fig. 1) (51). Driven by advancements in gene editing tools, researchers are now targeting specific genes in *K. phaffii* to improve the performance of desired traits and enhance fermentation efficiency. Research has shown that constructing a constitutive expression system with the *P_GAP_* and GUT1 homologous arms and using the *GUT1* gene as a targeting integration site allows for efficient screening of *K. phaffii* strains with high expression of bacterial fumonisin esterase enzyme (CFE) from a single-copy integrant (52). Building upon this concept of targeted modification, researchers have explored altering metabolic pathways to increase the production of various target compounds. For instance, Fina et al. (53) enhanced the production of 3-hydroxypropionic acid (3-HP) by introducing malonyl-CoA as a metabolic precursor into the 3-HP pathway of *K. phaffii* and overexpressing acetyl-CoA carboxylase (ACC), which catalyzes the formation of malonyl-CoA from acetyl-CoA. This highlights the immense potential of metabolic engineering for optimizing target compound production. Moreover, De et al. (54) expressed the *Bos taurus* lactate dehydrogenase (*LDH*) gene in the genome of *K. phaffii* X-33, generating a strain capable of producing L-lactic acid and utilizing glycerol as its sole carbon source. The lactic acid production of this strain increased by 20% under lower oxygen (O_2_) conditions, with the best strain achieving a lactic acid yield of approximately 0.7 g/g. Although the achieved titer still lags behind that of native lactic acid bacteria, this work demonstrated for the first time that *K. phaffii* presents a viable alternative for lactic acid production, offering significant feedstock cost reductions. Collectively, these results provide further evidence that targeted regulation of the metabolic processes in *K. phaffii* can substantially enhance the production efficiency of various high-value compounds.

After *K. phaffii* enters the methanol induction phase, methanol metabolism begins. Specifically, methanol metabolism occurs through alcohol oxidase (AOX), which oxidizes methanol to formaldehyde and hydrogen peroxide. The hydrogen peroxide is then decomposed into O_2_ and water by catalase (CAT). Formaldehyde is further assimilated in the peroxisomes through dihydroxyacetone synthase (DAS) in the xylulose monophosphate (XuMP) pathway, producing GAP. This G3P then enters the EMP pathway to form pyruvate, which enters central carbon metabolism and provides metabolic products to sustain cell growth. In addition to this assimilation pathway, formaldehyde is also catabolized to carbon dioxide (CO_2_), producing cytosolic NADH, which is then oxidized in the respiratory chain to generate energy through the catalysis of formaldehyde dehydrogenase (FLD), S-formylglutathione hydrolase (FGH), and formate dehydrogenase (FDH) (Fig. 1) (19, 55, 56). Feng et al. (57) engineered the strain via the co-expression of *K. phaffii* FLD, *S. cerevisiae* NADH kinase (Pos5), and the *K. phaffii* 2-oxoglutarate transporter (Odc1). This strategy aimed to promote formaldehyde assimilation, ensure optimal NAD^+^/NADH redox balance, and subsequently improve methanol utilization efficiency by 10%. This manipulation channeled increased carbon flux into central metabolic pathways, effectively boosting the specific productivity of xylanase B and resulting in a 101.8% increase in specific productivity and a 93.2% increase in volumetric productivity relative to the control. Given the dual pathways for formaldehyde metabolism, Guo et al. (58) sought to optimize the methanol assimilation pathway. This was accomplished through the knockout of glucose-6-phosphate isomerase (G6PI) in the XuMP cycle to optimize methanol assimilation and through modulation of pyruvate decarboxylase (PDC) activity to block the byproduct pathway for malate synthesis. This reduced NADH consumption and increased the availability of substrates and energy reserves for malate production.

Furthermore, comparative analyses based on transcriptomics and metabolomics showed that the order of gene downregulation in the methanol dissimilation pathway of *K. phaffii* was proportional to the downregulation of the oxidative phosphorylation pathway. More specifically, when the dissimilation pathway is hindered, the oxidative phosphorylation and assimilation pathways also weakened, leading to a low energy supply and defects in carbon fixation, ultimately affecting biomass accumulation and gene replication efficiency. Among these effects, formaldehyde-induced DNA-protein cross-linking may lead to transcriptional upregulation of the proteasome and autophagy (59). Interestingly, cellular responses to formaldehyde toxicity are varied and complex. In contrast, Berrios et al. found that cells could meet their energy needs by rearranging the pathway flux of EMP and the TCA cycle, thus protecting themselves from the toxic effects of formaldehyde (60).

Notably, optimizing carbon metabolic pathways can also be used to engineer high-yielding *K. phaffii* strains and further enhance the production and productivity of heterologous proteins and small molecules. After methanol is assimilated to produce pyruvate, it is converted to acetyl-CoA by the pyruvate dehydrogenase (PDH) complex. This acetyl-CoA then enters the mevalonate (MVA) pathway to produce isopentenyl pyrophosphate (IPP) and dimethylallyl pyrophosphate (DMAPP). Finally, IPP and DMAPP condense to form farnesyl pyrophosphate (FPP) (Fig. 1). Xu et al. (61) obtained a high-α-farnesene-producing *K. phaffii* strain, XF22, by using the *P_GAP_* to enhance the overexpression of key genes in the MVA pathway, increasing the supply of cytosolic acetyl-CoA, and reducing the accumulation of competing products of FPP synthesis.

##### Optimization of nitrogen metabolism

**Fig 2:**
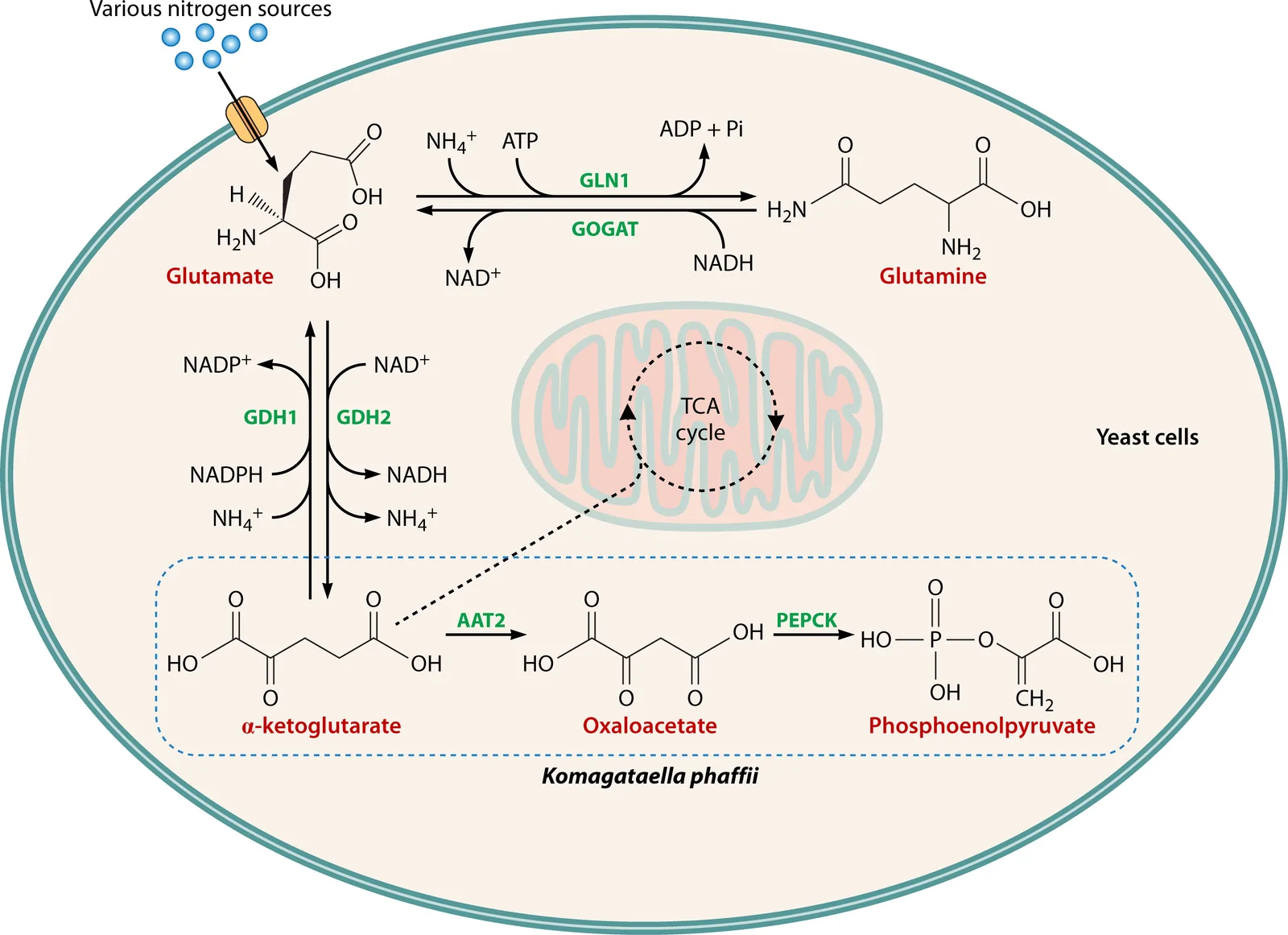
Central pathways for nitrogen metabolism. GDH1, NADPH-dependent glutamate dehydrogenase; GLN1, glutamine synthetase; GOGAT, glutamine-oxoglutarate aminotransferase; GDH2, NAD-dependent glutamate dehydrogenase; AAT2, aspartate aminotransferase 2; PEPCK, phosphoenolpyruvate carboxykinase.

Nitrogen metabolism is essential for the synthesis and degradation of amino acids and proteins. Yeast cells initially convert various nitrogen sources to glutamate and glutamine, which subsequently feed into the central nitrogen metabolism (CNM) pathway. Under the catalysis of NADPH-dependent glutamate dehydrogenase (GDH1), ammonia directly couples with α-ketoglutarate to form glutamate (62). Subsequently, glutamine synthetase (GLN1) catalyzes the formation of glutamine from ammonia and glutamate (63, 64). Glutamine can then produce glutamate again under the catalysis of glutamine-oxoglutarate aminotransferase (GOGAT). Additionally, glutamate can be degraded into α-ketoglutarate and ammonia by NAD-dependent glutamate dehydrogenase (GDH2) (Fig. 2) (65, 66). Studies have shown that regulating nitrogen metabolism by individually overexpressing GDH1 or GLN1 can increase the total nitrogen content and protein levels in the strain (67). Liu et al. (68) demonstrated that glutamate supplementation modulated the metabolic flux of metabolites and energy at key nodes of central metabolism in *K. phaffii*, resulting in a 32% enhancement in β-galactosidase yield. Furthermore, analysis via metabolomics and transcriptomics by Hannes et al. (69) revealed that overexpressing amino acid metabolism-related genes enhances recombinant protein yields; specifically, overexpression of the transcription factor GCN4 amplified the titer of the model protein secreted carboxylesterase (CES) by 2.6-fold.

Interestingly, *K. phaffii* exhibits a unique glutamate utilization pathway, enabling it to utilize amino acids as the sole source of both nitrogen and carbon. During carbon starvation, *K. phaffii* induces the synthesis of cytosolic GDH2, aspartate aminotransferase 2 (AAT2), and phosphoenolpyruvate carboxykinase (PEPCK) via autophagy. This triggers the post-transcriptional regulatory circuits for these enzymes, whereby the mRNA translation of GDH2 and PEPCK is selectively activated upon induction by their respective metabolic substrates, glutamate and oxaloacetate, thereby fulfilling the cell’s metabolic requirements (70, 71).

However, not all nitrogen sources are utilized efficiently. Nitrogen catabolite repression (NCR) is the regulatory mechanism by which yeast silences the expression of genes involved in the catabolism of less-preferred nitrogen sources (72). In yeast, when the preferred carbon source, glucose, is abundant, the EMP and TCA cycles supply the necessary precursor metabolites and energy required for nitrogen metabolism. Consequently, the NCR pathway is fully activated, leading the cell to prioritize the utilization of readily assimilable nitrogen sources. Upon the addition of a preferred nitrogen source to the growth medium, the transcription of permease genes, which encode proteins responsible for utilizing less preferred nitrogen sources, is suppressed, and their corresponding products are degraded (73). The NCR pathway has been extensively studied in *S. cerevisiae*, but less so in *K. phaffii* (74). In *S. cerevisiae*, NCR is primarily mediated and regulated by four GATA transcription factors, including glutamine metabolism protein (*GLN3*), GATA-type zinc finger DNA-binding motif protein (*GAT1*), allantoin pathway protein (*DAL80*), and GATA zinc finger factor 3 (*GZF3*)(75).

Despite this research gap, investigating nitrogen limitation in yeasts offers the potential for fermentation enhancement. Some evidence suggests that nitrogen limitation in yeast triggers the use of significant metabolic and translational reserves, enabling a rapid response to alterations in the growth environment. For example, glucose metabolism has over 80% reserve capacity, metabolic super pathways have over half of their metabolic capacity in reserve, gene expression exhibits over half of its translational capacity in reserve (mostly for translating metabolic proteins), and ribosomes have a 30% reserve capacity of sub-stoichiometric ribosomal proteins (76). Thus, further research is warranted to elucidate the regulatory pathways governing the optimization of carbon, nitrogen, and energy metabolism in *K. phaffii*, offering potential insights for maximizing the efficiency of high-density *K. phaffii* fermentation.

##### Optimization of energy metabolism

It has been reported that the production yield of recombinant proteins and small molecules is closely related to the availability of energy. When yeast cells overexpress recombinant proteins or small molecules and experience metabolic burden, not only are their carbon and nitrogen metabolism affected, but their energy metabolism is also significantly impacted (77, 78). Thus, in turn, alterations in the redox cofactor balance can influence the cell growth rate, thereby further affecting the production yield of recombinant proteins and small molecules (79). Therefore, increasing NADPH flux and maintaining a favorable NADPH/NADP^+^ ratio are key long-term metabolic engineering objectives (80).

The pentose phosphate (PPP) pathway is essential for maintaining the cellular NADPH/NADP^+^ balance (81). Glucose-6-phosphate dehydrogenase (ZWF1) and 6-phosphogluconolactonase (SOL3), which serve as rate-limiting enzymes in the PPP, exhibit synergistic effects. Co-expressing these enzymes causes a significant increase in PPP flux, thereby supplying sufficient energy for the effective accumulation of recombinant proteins and small molecules (82). Altering the redox balance in *K. phaffii* by overexpressing *POS5* from *S. cerevisiae* leads to changes in flux distribution in its central carbon metabolism. Targeting *POS5* to the cytoplasm led to a 2-fold increase in the production of recombinant antibody fragments (Fab). The copy number of Pos5 showed a positive correlation with both the NADPH/NADP^+^ ratio and Fab productivity (83). Reconstruction of the NADPH and ATP biosynthesis pathways to construct a high α-farnesene-producing *K. phaffii* strain involved optimizing key enzymes in the PPP, namely ZWF1 and SOL3, to improve NADPH supply. This was followed by low-intensity expression of heterologous Pos5 and further overexpression of endogenous adenine phosphoribosyltransferase (APRT), along with inactivation of NADH-dependent dihydroxyacetone phosphate reductase (GPD1), to increase both ATP and NADH levels. Ultimately, this resulted in an α-farnesene-producing *K. phaffii* strain with a 41.7% increase in productivity (84).

### Dynamic metabolic regulation

In the past few decades, with researchers’ continued advancement of genetic tools, systems biology techniques, and synthetic biology technologies, traditional static metabolic regulation strategies—such as gene knockout, promoter optimization, 5′ untranslated region (5′ UTR) modification, and protein engineering—have become insufficient to meet the demands for microbial metabolite production (85–87). In contrast to statically controlled engineered strains, which are sensitive to changes in the growth environment, dynamic control can sense environmental changes in real time and promptly adjust the flux of metabolic pathways, thereby improving the stability and production efficiency of strains in synthesizing target metabolites (88). Currently, developed dynamic control strategies include two-phase metabolic switching and continuous metabolic control (89).

#### Two-phase metabolic switching

Two-phase metabolic switching involves a fast growth phase and a product formation phase. During the fast growth phase, the wild-type phenotype of the strain is restored to achieve optimal cell growth rate. In the product formation phase, the focus is on maximizing the flux through the product pathway (90). The transition between the two phases is controlled by a metabolic switch, requiring sufficient stimulation to reach a designated threshold before switching occurs (91). This bistable metabolic switch activates and maintains the stability of the production state (92, 93). Research indicates that adjusting dissolved O_2_, inducer concentration, and pH can reliably control the completion of the transition between these two phases (94).

#### Continuous metabolic control

Continuous metabolic regulation enables cells to autonomously perceive signals from both the external environment and internal metabolic states and dynamically modulate the expression of key enzymes, thereby redirecting metabolic flux and enhancing the yield of target products (95). Metabolic pathways are dynamically regulated via signal sensors and control switches to achieve sustained metabolic control. This involves promoter selection, feedback control of enzyme expression in response to endogenous signals, or the coordinated inhibition of upstream pathways that produce toxic intermediates and activation of downstream conversion pathways, thereby achieving metabolic auto-balancing and preventing the accumulation of toxic intermediates (96–101).

#### Sensors and control switches

A dynamic metabolic control process generally consists of a sensor that detects a signal and a control switch that responds to the signal and converts it into a specific metabolic system output. In this context, dynamic regulation strategies based on signal changes are particularly important in microbial metabolic engineering. Examples of such signals include chemical inducers, nutrient sensing, O_2_, temperature, light, and pH (97, 102). In *K. phaffii*, by controlling O_2_-limited conditions and specific growth rate, the specific product formation rate of *Candida rugosa* lipase 1 (Crl1) is twice that under normal O_2_ conditions (103).

Furthermore, optimization of the sensor signals and control switches that respond to metabolic system outputs is also feasible. In *Klebsiella thermophila*, L-aspartate α-decarboxylase (ADC) catalyzes the decarboxylation of L-aspartate to produce β-alanine. By overexpressing aspartate dehydrogenase to increase ADC gene dosage and further enhancing the supply of the aspartate precursor via phosphoenolpyruvate (PEP) carboxylase, a β-alanine concentration of 5.6 g/L was achieved through fed-batch cultivation (104). Zhang et al. replaced the promoters of key glycolytic enzyme genes with a glycerol-inducible promoter, enabling a dynamic switch from cell growth to inositol production, resulting in a 4.9-fold increase in inositol yield (105). However, most reports concerning dynamic metabolic control strategies are focused on *E. coli*, with few related studies on *K. phaffii*. This may be due to the complex transcriptional regulatory mechanisms of *K. phaffii* and the insufficient application of interdisciplinary approaches.

### Metabolic model

Currently, the application of systems biology also plays a crucial role in characterizing cell metabolism and guiding key targets for metabolic engineering. Systems biology integrates high-throughput omics data and utilizes computational modeling and analysis to achieve specific desired cellular systems (106). Based on the genome sequencing of *K. phaffii* (42, 44), genome-scale metabolic models (GEMs) of *K. phaffii* have now been established (107–109). The early construction of GEMs was based on strains DSMZ 70382 and GS115, respectively, resulting in two similarly scaled models named PpaMBEL1254 and iPP668. Although their scales were similar, iPP668 further expanded and refined the methanol utilization pathway and its peroxisome compartment (107, 109). Subsequently, the iLC915 model, constructed from the GS115 strain, represented a significant leap forward. It accomplished the pioneering integration of the complete methanol utilization pathway, peroxisome compartment, glycosylation precursors, and mixed carbon sources utilization into GEM (108). To further enhance the comprehensiveness of the model, researchers constructed a new consensus model, iMT1026, by comparing, integrating, and updating the aforementioned GEMs. This model not only standardized metabolite nomenclature but also encompassed critical metabolic processes, such as protein glycosylation pathways, lipid metabolism, and energy metabolism (49). Research indicates that by analyzing the biomass composition of *K. phaffii* cultivated under different growth rates and with various carbon sources, the functionality of the iMT1026 model was updated, which significantly enhanced its predictive capability for growth on glycerol and methanol as sole carbon sources. Subash et al. (110) constructed the iAUKM model by merging iMT1026 with a draft model generated from the KEGG database. iAUKM can predict target genes for overexpression or knockout within the TCA cycle, EMP, and PPP pathways. When simulating overexpression targets for S-adenosyl-L-methionine (AdoMet), inorganic pyrophosphatase (IPP) was identified as an effective target, enabling the precise enhancement of recombinant protein synthesis efficiency. The experimental data showed that in media containing glucose and glycerol, AdoMet production increased by 16% and 14%, respectively (111).

However, current GEMs are primarily confined to steady-state metabolic analysis under static conditions, whereas dynamic conditions are often simulated using dynamic GSMs. The GSM, based on the dynamic flux balance analysis (dFBA) framework developed by Sánchez et al. (112), simulates the dynamic metabolic processes of *K. phaffii* under glucose-limited, aerobic, and fed-batch cultivation conditions via three iterative solving modules: kinetic, metabolic, and dynamic. Model-based prediction and validation revealed that knockout of the gene encoding methylenetetrahydrofolate dehydrogenase (MTHFD) increased the volumetric productivity of recombinant human serum albumin (HSA) by 6.3-fold (113).

Fueled by the increasing sophistication of machine learning, researchers are integrating computational tools with experimental work, departing from singular, traditional experimental methods. Relative to traditional modeling, machine learning significantly shortens the design cycle of metabolic pathways by uncovering subtle but interconnected patterns within data and integrating with the metabolic network of GEMs (114). Pentjuss et al. (115) developed a growth-coupled analysis algorithm to systematically explore the GEM of *K. phaffii*, enabling it to screen for key metabolic reactions affecting heme production. Compared with traditional methods, this method increased the biomass growth rate on glycerol by 50%, and growth on methanol approached the theoretical maximum.

### Optimizing chassis cells

*K. phaffii*, engineered as a chassis organism for sustainable biomanufacturing, produces heterologous proteins and small molecules. A suitable hemoglobin expression system was constructed by overexpressing a single copy of *P_AOX1_* and the transcription activator *MIT1*, and by deleting the aspartic protease *YPS1-1* gene to inhibit hemoglobin degradation. The heme biosynthesis pathway was reconstructed in the cytoplasm, and rate-limiting steps were enhanced, leading to a *K. phaffii* chassis strain with 37-fold higher productivity compared to the original strain. This improved strain is capable of producing high-activity porcine myoglobin, soybean hemoglobin, erythrocyte hemoglobin, and P450-BM3 (116). Further, a *K. phaffii* chassis strain optimized for the biosynthesis of L-tyrosine (L-Tyr) precursors was developed. This involved overexpressing feedback-insensitive variants of 3-deoxy-D-arabino-heptulosonate-7-phosphate synthase (ARO4K229L) and chorismate mutase (ARO7G141S) in the shikimate pathway. This metabolic engineering strategy provides an efficient approach for *K. phaffii* to produce increased amounts of precursors for aromatic compounds, including resveratrol, naringenin, and chlorogenic acid, from glycerol byproducts (117).

Currently, the vast majority of research on *K. phaffii* chassis strains revolves around the GS115 and X-33 strains. Although the NRRL Y-11430 strain and its derived industrial strains GS115 and X-33 were under commercial restriction, with the expiration of relevant patents, comparative genome sequencing revealed that the openly available NCYC 2543 strain is highly similar to NRRL Y-11430. Furthermore, by introducing a single base pair deletion in the *HOC1* gene of the NCYC 2543 strain, the NCYC 2543 HOC1tr-1 mutant was generated; this mutation significantly improved its transformation efficiency and the secretion capacity of some proteins. Based on these findings, researchers developed a strain named OPENPichia and provided a set of synthetic modular expression vector toolkits based on a free end-user distribution license, thus making royalty-free commercial production possible (118). This work not only pioneered new avenues for *K. phaffii* in metabolic engineering and synthetic biology but also established a robust foundation for its widespread deployment in the future bio-manufacturing industry.

### Recombinant protein production

#### Glycosylation engineering

Glycosylation is one of the most complex and prevalent post-translational modifications (PTMs) of proteins and exerts a determinative influence on protein stability, activity, secretion, and immunogenicity. Glycosylation in eukaryotes is primarily classified into N-linked glycosylation and O-linked glycosylation (119). N-linked glycosylation occurs at asparagine residues and is initiated by the attachment of N-acetylglucosamine (GlcNAc) to the consensus sequence Asn-X-Ser/Thr (where X ≠ is proline [Pro]) in the ER, after which it can be elongated into complex, hybrid, or high-mannose forms (120, 121). O-linked glycosylation involves the direct linkage of GalNAc or mannose (Man) to serine/threonine (Ser/Thr) residues, lacks a conserved motif, is predominantly processed in the Golgi apparatus, and results in diverse core structures (122–124).

Compared to prokaryotic organisms (such as *E. coli* and *Bacillus*), which inherently lack glycosylation capabilities, and to the production bottlenecks brought by the high cost, susceptibility to contamination, and intricate regulatory networks of mammalian cells (CHO cells), *K. phaffii*, with both rapid growth and ease of genetic tractability, has become an ideal host for producing recombinant proteins and small molecules. Therefore, glycoengineering strategies to modify *K. phaffii* by eliminating its endogenous high-mannose-type glycosylation modification and introducing the human glycosylation pathway are critically important for enhancing the competitiveness of *K. phaffii* in the production of recombinant proteins and small molecules (21).

Over the past two decades, glycoengineering of *K. phaffii* has achieved significant milestones: the knockout of genes such as *OCH1* blocks the high-mannose extension, while the introduction of α-1,2-mannosidase generates Man_5_GlcNAc_2_, yielding the commercially viable GlycoSwitch-engineered strains (125). Further advancements involved expressing enzymes like N-acetylglucosaminyltransferases I/II (GnT-I/II) and galactosyltransferases (GalT) to achieve humanized complex-type N-glycans (e.g., Gal_2_GlcNAc_2_Man_3_GlcNAc_2_). Moreover, some strains achieved terminal biorthogonal sialylation via a single plasmid expressing five genes: UDP-GlcNAc 2-epimerase/N-acetylmannosamine kinase (GNE), N-acetylmannosamine-9-phosphate synthase (SPS), CMP-N-acetylneuraminic acid synthase (CSS), CMP-sialic acid transporter (CST), and α-2,6-sialyltransferase (ST) (126–128). Additionally, by knocking out the *ALG3* gene in *ΔOCH1 K. phaffii* strains, the synthesis of the Lipid-Linked Oligosaccharide (LLO) precursor in the ER is blocked, forming a truncated LLO, which can directly yield the Man_3_GlcNAc_2_ core structure, thereby circumventing interference from endogenous Golgi mannosyltransferases and enhancing glycan homogeneity (129, 130). For proteins where glycan function is dispensable, the heterologous expression of Endo-β-N-acetylglucosaminidases (ENGases) (e.g., EndoH, EndoT) in *K. phaffii* can cleave the β-1,4-glycosidic bond of high-mannose or hybrid N-glycans, retaining only a single GlcNAc residue (131, 132).

In O-glycosylation, yeast engineering is mainly achieved by reducing specific O-glycan chains or realizing humanized O-glycan synthesis. In *K. phaffii*, knocking out the *protein Mannosyltransferase 1* (*PMT1*) gene significantly reduces O-glycosylation of proinsulin precursors; further deletion of *PMT2* can potentially shorten the glycan chain length, but cytotoxicity from multiple knockouts must be avoided (133, 134). Alternatively, PMT inhibitors, such as rhodanine-3-acetic acid, can be employed during the induction phase of protein expression (135). Reducing O-glycan chains can also be achieved by expressing specific mannosidases; for instance, expressing α-1,2-mannosidase in the Golgi apparatus can trim O-glycan chains to a monomannose structure. *In vitro*, complete removal of O-linked mannose can be accomplished using enzymes such as Jack Bean α-1,2/3,6-mannosidase or lysosomal mannosidases (136–138).

In *K. phaffii*, humanized O-glycosylation engineering primarily targets two major types: mucin-type and α-dystroglycan-type O-glycans. In the mucin-type pathway, protein O-mannosyltransferases (the heterodimeric *PMT1/PMT2* or homodimeric *PMT4*) catalyze the transfer of a Man residue from the dolichol-phosphate-mannose (Dol-P-Man) donor to Ser/Thr residues of target proteins via an α1-O-glycosidic linkage, forming Manα1-O-Ser/Thr. This initial structure is subsequently elongated in the Golgi apparatus by α-1,2-mannosyltransferases (Ktr1/Kre2) and α-1,3-mannosyltransferases, resulting in oligomannose chains (Man_2–9_) containing 2–9 mannose residues. These chains can be targeted by mannose-binding proteins, such as the macrophage mannose receptor (MR), dendritic cell-specific intercellular adhesion molecule-3-grabbing non-integrin (DC-SIGN), and mannose-binding lectin (MBL). The α-dystroglycan-type O-glycosylation chain strategy is based on *K. phaffii* strains deficient in phosphorylated and β-mannose structures. By overexpressing the murine protein-O-linked-mannose β-1,2-N-acetylglucosaminyltransferase I (PomGnT-I), galactosyltransferase (GalT), and sialyltransferase (SiaT), sialylated O-glycan chains are constructed, achieving a 61% humanization rate of O-glycans in a TNFR2:Fc fusion protein (136, 139).

The camelid variable domain of the heavy-chain-only (VHH) antibodies, as a new class of therapeutic proteins, has advantages such as small molecular size, strong antigen-binding specificity, and high stability. These give them broad application prospects in fields such as tumor-targeted therapy, viral neutralization, and cell-targeted delivery. However, in their natural state, VHH antibodies have very few glycosylation sites, and most of these are located in the antigen-binding complementarity-determining regions (CDRs), which can easily affect antigen binding. By screening potential glycosylation sites (e.g., Q14N-P15A-G16T, G27N-P30T) in the VHH antibody framework regions (non-CDR areas) and expressing the antibodies in the *K. phaffii* GlycoSwitchM5 strain, homogeneous N-glycosylated products mainly composed of Man_5_GlcNAc_2_ were obtained. The site-specific glycosylation occupancy rate reached 71%–96%. Moreover, the modified VHH antibodies still maintained nanomolar antigen-binding affinity and also achieved glycan-dependent targeted uptake by macrophages (140).

The anti-human epidermal growth factor receptor 2 antibody (anti-HER2 antibody) is a first-line therapeutic drug for treating HER2-positive breast cancer and gastric cancer. However, the native O-mannosylation in *K. phaffii* can lead to increased antibody immunogenicity and protein degradation. Moreover, existing glycosylation engineering efforts have mostly focused on N-glycosylation and lack strains that simultaneously optimize both N- and O-glycosylation. Starting from the low-mannose N-glycosylation-engineered strain GJK01, further knockout of *PMT1* resulted in the construction of the dual-optimized strain GJK11-HL (*ΔOCH1*, *ΔPMT1*). This reduced the O-mannose content on the antibody from 14 to 5 sites per molecule, decreased the heavy chain degradation bands by 65%, increased expression yield by 24%, and did not cause growth defects (141).

The tetanus toxin fragment C (TetC) is a non-toxic fragment derived from *Clostridium tetani* and consists of 451 amino acids. It serves as the core subunit of the tetanus toxoid vaccine. Its secretion efficiency and stability are jointly influenced by the number and location of N-glycosylation sites. Five natural N-glycosylation sites on TetC were predicted using the NetNGlyc 1.0 Server. Eight mutants were constructed via site-directed mutagenesis. It was found that the 4Gly mutant, in which the natural site N280Q was deleted while the other four natural sites were retained, exhibited the highest secretion yield. Conversely, glycoengineering that introduced a new N-glycosylation site at position 320 further inhibited secretion (142).

#### Endoplasmic reticulum (ER) engineering

Although *K. phaffii* has become one of the preferred eukaryotic systems for recombinant protein expression, its high-yield capability is still limited by multiple non-glycosylation factors. The ER, as the primary site for protein synthesis and subsequent folding, often experiences ER stress upon high-level expression of heterologous GlycoVHH—optimal sites for introducing N-glycans on the camelid VHH antibody scaffold and for macrophage delivery—leading to misfolding, aggregation, or degradation. To alleviate this limitation, strategies such as co-expression of molecular chaperones (e.g., the ER-resident Hsp70 family protein *KAR2P*) and regulation of the unfolded protein response (UPR) pathway (e.g., overexpression of the key transcription factor *HAC1p*) have been widely adopted, significantly improving secretion efficiency and protein integrity (143, 144).

Recombinant proteins produced commercially in *K. phaffii* are widely used in biopharmaceuticals and diagnostic reagents. However, the secretion titer of such proteins can vary by tens to hundreds of times, and high-level expression is often limited by a translocation bottleneck. The use of strong promoters or high-copy expression leads to a synthesis rate of nascent polypeptide chains that exceeds the capacity of the post-translational translocation channel, causing polypeptide aggregation and UPR activation. Approximately 60% of the product is degraded via the endoplasmic reticulum-associated degradation (ERAD) pathway, and merely increasing gene copy number can actually reduce the secretion rate. By replacing the *MFα* signal peptide with co-translational signal peptides such as *OST1* and *YAP3* to reduce intracellular retention, and by co-expressing components of the SRP/Sec61 complex (e.g., *SRP54P* and *SEC61P*), the secretion yield of Fab can be increased 1.5- to 3-fold. Concurrently, reducing gene copy number or using a weaker promoter can decrease the translocation burden (144).

On this basis, by employing push-pull strategies on both the cytoplasmic and ER sides, along with the coordinated regulation of factors participating in the ER Hsp70 chaperone cycle, the translocation, folding, and secretion of recombinant proteins during the translocation step can be increased. This resulted in secretion levels of antigen-binding fragments (Fab) and single-chain variable fragments (scFv) exceeding 1.3 g/L, representing a 5-fold increase compared to the original levels (145).

Recombinant human hyaluronidase PH-20 (rhPH-20) is widely used in pharmaceutical, food, and cosmetic applications. However, extraction from animal sources poses safety risks, and its complex N-glycosylation pattern relies on the eukaryotic post-translational modification system of *K. phaffii* to maintain activity. Yet, expression in *K. phaffii* faces issues such as extremely low yield, a high misfolding rate due to hyperglycosylation, and protein intracellular aggregation, and oxidative stress triggered by the traditional *MFα* signal peptide-mediated post-translational translocation. By constructing an *OST1-PROΑ* hybrid signal peptide to switch the translocation mode to co-translational, overexpressing the core subunit of the signal recognition particle (SRP) to broaden the translocation pathway, and co-overexpressing *SCHAC1P* to alleviate ER stress and oxidative damage, a high expression level of 19.8 U/mL was finally achieved in a 5 L fed-batch fermentation (146).

The humanization of N-glycan structures on glycoproteins also relies on translocation and processing within the ER. Truncation of the LLO leads to the absence of glucose residues, which impairs the protein folding quality control mechanism that depends on them. The exposed α1-6-linked mannose is easily recognized by Yos9p, triggering erroneous ERAD. Moreover, a decrease in glycosylation site occupancy exacerbates the aggregation of unglycosylated peptide segments. Introducing a co-translational signal peptide can accelerate the entry of nascent peptides into the ER to reduce aggregation. Overexpressing a single-subunit oligosaccharyltransferase (OST) or *ALG6* can improve glycosylation efficiency. Knocking out *HRD1* or *YOS9* can block the erroneous ERAD pathway. Ultimately, this achieves a balance between glycan humanization and high secretion efficiency (147).

#### Examples of recombinant proteins

##### Nutrients

Soy leghemoglobin (LegH) serves not only as a colorant and flavoring ingredient in food products but also as an iron provider for the body, holding considerable application potential in plant-based meat analogs and nutraceuticals. The core challenge in its heterologous expression lies in the need to simultaneously achieve high secretion titers and high heme incorporation rates. Furthermore, the relatively low genomic integration efficiency in *K. phaffii* often leads to unstable expression of the target gene. Recently, researchers have engineered *K. phaffii* strains capable of high-level expression of LegH through a series of engineering efforts. By integrating the *LEGH* gene into *K. phaffii*, increasing the gene copy number, overexpressing all gene combinations of the heme biosynthesis pathway—including *HEM1*–*4* and *HEM12–15*—under the regulation of the *P_AOX1_*, and supplementing the *KU70* gene, the highest reported secretion level of leghemoglobin was achieved in fed-batch high-cell-density fermentation. The LegH titer reached 3.5 g/L, with a heme-binding rate of 93% (148).

##### Drug

Diosgenin, a plant-originated secondary metabolite, is extensively employed as a precursor for steroidal drug synthesis within the pharmaceutical industry (149). In contrast to the traditional method of producing diosgenin through acid hydrolysis, the recombinant β-glucosidase FBG1 expressed in *K. phaffii* can efficiently biotransform trillin (diosgenin glucoside) into diosgenin. This recombinant enzyme is characterized by high purity and stability, the absence of environmental pollution, and low cost, offering potential advantages in sustainable production and environmental protection (150). The primary challenges in applying this enzyme stem from its extremely low yield from natural sources and issues such as imbalanced metabolic flux distribution during heterologous expression in *K. phaffii*, which readily restrict efficient secretion. Heterologous expression of the *FBG1* gene from *Fusarium* sp. CPCC 400709 in *K. phaffii* GS115, combined with the development of a co-stat fed-batch high-cell-density fermentation strategy combining constant exponential feeding rate (μ-stat) and constant methanol concentration (m-stat), significantly enhanced the high-level expression of recombinant FBG1. The maximum volumetric activity reached 89 × 10⁴ U/L, and the maximum specific activity was 90 × 10² U/g (151).

Interferon α2b (IFNα2b) is a cytokine that specifically interacts with the IFN α/β receptor, activating the JAK-STAT signaling pathway and inducing biological functions such as immune modulation, antiproliferation, and antiviral activity. It is vital to the treatment of viral infections (152), human immunodeficiency diseases (153), various cancers (154), and COVID-19 (155). Traditional fermentation processes are prone to host metabolic imbalance due to fluctuating methanol concentrations, and real-time monitoring of critical parameters is challenging. Currently, recombinant human interferon α2b (huIFNα2b) expressed in *K. phaffii* was produced using a model-based adaptive proportional-integral (PI) control framework. The framework utilizes a fermentation calorimeter, a CO₂ gas analyzer, and a methanol sensor to monitor the residual methanol concentration in the culture medium in real-time. By employing a fed-batch fermentation process, the methanol concentration was dynamically regulated to optimize huIFNα2b production. This resulted in optimal huIFNα2b production at a methanol concentration of 3 g/L, with a maximum titer of 244.34 mg/L and a maximum productivity of 0.31 mg/g·h (156).

##### Other

Agricultural residues such as rapeseed straw and corn stover are rich in cellulose and lignin. However, due to the complex physicochemical structures of cellulose and lignin, ruminants have difficulty efficiently digesting and absorbing the nutrients contained in these materials. Therefore, the efficient degradation of these components is a core step for achieving the valorization of agricultural residues. Recombinant laccase (LeLac), a key functional enzyme involved in lignin degradation, offers advantages such as mild reaction conditions and the absence of secondary pollution, making it well aligned with the development trends in sustainable manufacturing. However, native laccases generally exhibit low degradation efficiency toward complex lignin substrates and poor stability, which limits their suitability for large-scale applications. Heterologous expression of LeLac from *Lentinula edodes* in *K. phaffii* can cleave bonds in lignin substrates within rapeseed straw, providing sustainable potential for bioenergy and waste utilization in agriculture (157). For example, expression of the *Pleurotus ostreatus* laccase gene *LAC-2* in the *K. phaffii* strain X-33 resulted in a LeLac with an 18.36% degradation rate of lignin in corn stover (158). As another example, optimization and integration of the laccase gene *LAC-1* from *Coriolopsis trogii* strain Mafic-2001 into *K. phaffii* strain X-33, combined with process optimization, yielded a recombinant laccase with lignin degradation rates of 24.43%, 50.24%, and 55.49% for palm kernel cake matrix, rice straw, and corn stover, respectively (159).

### Small-molecule production

#### Regulating synthetic pathways

Regulating the biosynthetic pathways of *K. phaffii* is one of the primary methods for synthesizing various small molecules. Isobutanol represents a promising advanced biofuel and versatile platform chemical. It offers distinct advantages over conventional ethanol, including superior energy density, reduced hygroscopicity, and direct compatibility with existing fuel infrastructure, as well as its utility as a precursor for synthesizing diverse high-value chemicals (160). However, in the natural metabolic pathway of *K. phaffii*, the intermediate 2-ketoisovalerate is more inclined to flow toward inherent amino acid synthesis, leading to low efficiency in diverting flux to the 2-keto acid degradation (Ehrlich) pathway. Moreover, optimizing a single pathway alone easily causes metabolic imbalance (161). Overexpressing genes in the Ehrlich pathway that add the intermediate 2-ketoisovalerate (KIV), along with upregulating a fraction of the L-valine biosynthesis pathway, can further increase isobutanol production (102, 162).

Furthermore, overexpression of an alcohol O-alkyltransferase with a wide substrate range results in the synthesis of isobutanol and various higher branched-chain alcohol acetates. Isobutyl acetate possesses a fresh, fruity aroma, serving as a key ingredient in the food, cosmetic, and fragrance industries, and is also utilized as an industrial solvent (163). However, conventional strategies involving the integrated expression of alcohol O-acyltransferases often suffer from low catalytic efficiency. Isobutyl acetate possesses a fresh, fruity aroma, serving as a key ingredient in the food, cosmetic, and fragrance industries, and is also utilized as an industrial solvent. However, conventional strategies involving the integrated expression of alcohol O-acyltransferases often suffer from low catalytic efficiency. Among these, isomeric expression of an alcohol O-acyltransferase increased isobutyl acetate production 9-fold compared to the integrative expression (164).

Geraniol, a high-value monoterpenoid compound, finds extensive applications in flavors and fragrances, pharmaceuticals, and agrochemicals, representing significant economic potential (165). However, the heterologous synthesis of geraniol in *K. phaffii* faces issues such as significant differences in the compatibility of heterologous enzymes across different intracellular compartments (peroxisomes and cytoplasm) and enzyme activity being easily affected by the intracellular microenvironment. Overexpressing the genes (*ScERG10*, *ScERG13*, *ScERG12*, *ScERG8,* and *ScERG19*) encoding the remaining enzymes of the MVA pathway from *S. cerevisiae* in *K. phaffii* strains GERC8 and GERP8 to elevate the metabolic flux of the geraniol biosynthesis pathway led to a 2.2-fold and 3.0-fold increase for geraniol synthesis in the peroxisomes and cytoplasm, respectively (166).

#### Renewable substrates

Within the green biomanufacturing platform of *K. phaffii* for small-molecule production, commonly used renewable substrates include xylose derived from lignocellulosic biomass and CO₂. Sugarcane bagasse is a xylose-rich, economical, and renewable substrate with various sources, but *K. phaffii* has a limited capacity for assimilating natural xylose. Studies indicate that fermentation by *K. phaffii* at an optimized glucose-to-xylose ratio of 10% led to a xylitol titer of 86.6 g/L with a conversion rate of 0.75 g/g (167). A heterologous xylose isomerase pathway was introduced into the *K. phaffii* strain GS115 by overexpressing either the xylose isomerase gene from *Orpinomyces* spp. or an endogenous glucokinase gene. After 50 generations of continuous culture, this resulted in a recombinant *K. phaffii* strain with high xylose assimilation capacity and a cell yield nearly 2-fold higher than the starting strain GS115 (168). Furthermore, a metabolic pathway for producing ethylene glycol (EG) from xylose has been constructed in *K. phaffii*. EG is used to manufacture plastic polymers and serves as an antifreeze, hydraulic oil, and surfactant. However, issues such as metabolic pathway deviation and low xylose utilization efficiency exist. By introducing a novel xylonate dehydratase (xylD-HL), EG production was increased by 30%. Further, by heterologously expressing glyoxylate reductase (ALDR) and adjusting the initial glucose-to-xylose ratio to 50%, the maximum EG titer reached 1.31 g/L (169). Furthermore, xylose can also be utilized for the production of itaconate (2-methylidenebutanedioic acid), which finds extensive applications in the synthesis of resins, fibers, detergents, and bioactive compounds (170). Codon optimization of an overexpressed heterologous cis-aconitate decarboxylase can enhance itaconic acid production from xylose, with the engineered high-producing recombinant strain achieving a titer 28-fold higher than the starting strain (171).

Itaconate production can also be rendered autotrophic by incorporating a CO₂ fixation pathway. An alternative strategy involves the heterologous expression of the *CADA* gene from *Aspergillus terreus* and the reconstruction of the Calvin-Benson-Bassham (CBB) cycle. An autotrophic *K. phaffii* strain capable of using CO_2_ as the sole carbon source to produce itaconic acid was obtained. Further optimization of process conditions by balancing the co-expression of cis-aconitate decarboxylase (cadA) and the mitochondrial tricarboxylate transporter (mttA) resulted in an autotrophic *K. phaffii* strain with an itaconic acid yield of 2 g/L (172).

Furthermore, CO₂ can be efficiently converted into methanol via a Chemo-Biological Cascade Conversion (CBCC) system for subsequent product synthesis. The CBCC system integrates a thermocatalytic CO₂-to-methanol process with microbial fermentation. In the thermocatalytic stage, CO₂ is converted to methanol on a large scale over a CuZnAlC catalyst in the presence of H₂, achieving a CO₂ conversion rate of 8.5% and a methanol selectivity of 79% (173). *K. phaffii* utilizes methanol as a carbon source for the production of high-value chemicals. However, the production of 3-HP in *K. phaffii* currently faces challenges such as insufficient yield, metabolic flux imbalance, and accumulation of toxic intermediates. To address these issues, the pyruvate carboxylase gene *PYC2* was overexpressed to enhance precursor supply, the transporter genes *ESBP6/JEN1* were co-expressed to facilitate 3-HP export, and the *FDH1* gene was knocked out to reduce toxic intermediate formation. Coupled with strain optimization via CRISPR/Cas9, a titer of 27.0 g/L 3-HP was achieved in pH-controlled (pH 5) fed-batch cultivation, representing a 42% increase in concentration and over 20% improvement in productivity compared to the parental strain (174).

#### Examples of small molecules

##### Nutrients

Lycopene, a type of carotenoid, exhibits beneficial effects such as delaying cellular senescence and protecting cardiovascular health, and is extensively employed in the food, health supplement, and cosmetic fields. Conventional methods such as chemical synthesis or plant extraction suffer from drawbacks, including low product purity and high cost, making biosynthesis the preferred route for scalable production. However, this approach faces core challenges such as severe metabolic flux diversion and insufficient NADPH supply. By using and optimizing the CRISPR/Cpf1 system to knock out the *DPP1* and *LPP1* genes, which encode enzymes in competing pathways that produce farnesol, and by downregulating the *ERG9* gene, lycopene production was increased. Additional genomic integration of one extra copy of each of *CRTE*, *CRTB*, and *CRTI* further enhanced lycopene production by creating more pathway variants. Overexpression of *POS5* optimized NADPH supply in the energy metabolism pathway, providing sufficient metabolic flux for lycopene production. Transcriptomic and metabolomic analyses guided the overexpression of sterol regulatory element-binding protein (SREBP; Sre), which upregulated the MVA pathway to regulate lipid metabolism and promote lipid synthesis. High-cell-density fermentation in a 5 L fermenter led to a lycopene titer of 7.24 g/L and a dry cell weight of 75.48 mg/g in *K. phaffii*. This represents the highest lycopene production reported in *K. phaffii* to date (175).

##### Drug

Cordycepin (3′-deoxyadenosine), a compound first isolated from the caterpillar fungus *Cordyceps militaris* in the early 1950s (176), exhibits antibacterial, antiviral (177), antioxidant (178), anti-inflammatory (179), and antitumor activities (180). The traditional method of extracting it from *Ophiocordyceps sinensis* has drawbacks, such as low yield, high cost, and high resource dependence, making biosynthesis an inevitable choice for its large-scale production. Codon-optimized cordycepin biosynthetic genes *CNS1* and *CNS2* from *C. militaris* L5111, placed under the management of the *P_AOX1_* and *P_FLD1_* and integrated into the *K. phaffii* GS115 genome, have been shown to enhance cordycepin expression. Specifically, through fermentation optimization and transcriptomic analysis targeting methanol assimilation, peroxisome biogenesis, purine metabolism, the PPP pathway, and β-alanine metabolism, cordycepin production was increased. This optimized design led to a cordycepin titer of 2.68 ± 0.04 g/L, with a productivity of approximately 15.95 mg/(L·h) (181). Zhao et al. further engineered a high-yield strain of *K. phaffii* for cordycepin production. They focused on enhancing methanol assimilation, upregulating cellular energy metabolism, and increasing the availability of precursors such as adenosine and 3′-AMP via genetic engineering and biosynthetic pathway optimization. These combined efforts yielded cordycepin titers of 1,551.44 mg/L in shake flasks and 8.11 g/L in a 10 L fermenter (182). This success underscores the potential of *K. phaffii* for the production of valuable plant-derived compounds.

## CONCLUSIONS

*K. phaffii* has evolved into a core platform for heterologous proteins and small-molecule production in eukaryotic expression systems over the last 40 years, driven by its distinct capability to utilize the sustainable and renewable carbon source methanol. Through strategies such as metabolic flux analysis, precursor and energy supply enhancement, genome integration, key gene regulation, and dynamic metabolic control, its metabolic pathways have been systematically optimized to enhance its potential for industrial applications. Notably, in the realm of strain tool development, the advent of the openly accessible, royalty-free OPENPichia strain effectively breaks the commercial monopoly restrictions of traditional industrial strains. It provides a viable solution for the low-cost commercial production of heterologous proteins and small molecules in *K. phaffii*, thereby significantly enhancing the practical utility value of the strain (118).

To date, research advancement toward the metabolic engineering of *K. phaffii* has primarily relied on traditional genetic tools. The expeditious development of omics technologies and synthetic biology tools has also propelled *K. phaffii* metabolic engineering research. However, *K. phaffii* still faces some bottlenecks that hinder its ability to reach higher expression levels. Although the CRISPR/Cas9 genome editing tool has seen some application in *K. phaffii*, the available molecular tools are relatively limited compared to other model organisms. More tools need to be developed to meet future research requirements, such as promoters, terminators, and dynamic regulation tools. Secondly, the advancement of comprehensive GEMs is an essential step for *K. phaffii* metabolic engineering. Obtaining the large data sets required for genome-scale metabolic models and combining them with mathematical models to predict potential key gene targets within metabolic pathways can more comprehensively optimize overall metabolic pathways and enhance the final yield of target proteins. Furthermore, artificial intelligence (AI) has played an increasingly significant role in metabolic engineering. Compared to conventional experimentation, AI can effectively reduce the cost and time associated with experimental trial-and-error, particularly in the design and construction of new biosynthetic pathways, high-efficiency strains, and self-dynamic regulation systems (183–185).

## Data Availability

No data were used for the research described in the article.
